# Editorial: Advances and challenges in stroke therapy: A regenerative prospective

**DOI:** 10.3389/fnins.2022.1102119

**Published:** 2022-12-12

**Authors:** Syed Shadab Raza, Hassan Azari, Viola B. Morris, Aurel Popa Wagner

**Affiliations:** ^1^Laboratory for Stem Cell and Restorative Neurology, Department of Biotechnology, Era's Lucknow Medical College and Hospital, Era University, Lucknow, India; ^2^Department of Stem Cell Biology and Regenerative Medicine, Era's Lucknow Medical College Hospital, Era University, Lucknow, India; ^3^School of Podiatric Medicine, Barry University, Miami Shores, FL, United States; ^4^Division of Cardiology, Department of Medicine, Emory University School of Medicine, Atlanta, GA, United States; ^5^Department of Biochemistry, University of Medicine and Pharmacy of Craiova, Craiova, Romania; ^6^Department of Neurology, Vascular Neurology, Dementia and Ageing Research, University Hospital Essen, University of Duisburg-Essen, Duisburg, Germany

**Keywords:** stroke, stem cell therapy, nanomedicine, regenerative medicine, middle cerebral artery (MCA) occlusion, oxygen glucose deprivation (OGD)

Stroke is the one of the most common causes of death and the leading cause of disability globally (Owolabi et al., 2022). Over the previous three decades, global stroke incidence increased by 70%, prevalence increased by 85%, death increased by 43%, and disability-adjusted life years (DALYs) increased by 32%, with low and middle-income countries experiencing a greater increase in the burden of stroke than high-income countries (Feigin et al., 2019). Regenerative therapies, such as stem cell treatment and nanomedicines, have the potential to alleviate the neurological symptoms of cerebral stroke (Li and Sun, 2021; Prakash et al., 2021a,b, 2022; Wang et al., 2022; Raza et al., 2018). The current thematic issue emphasizes the significance of regenerative medicine research in stroke recovery, in addition to fully comprehending the pathology, with in-depth reviews and original research articles on a wide range of topics, including nanomedicines, miRNA, and stem cells for stroke recovery. In brief, the articles presented here cover a wide range of topics, from fundamental scientific investigations into the pathological mechanisms linked to cerebral stroke, to clinical trials. Current advances in regenerative biology, aided by novel concepts and approaches, may pave the way for new therapeutic developments in regenerative medicine. The current editorial highlights the findings of the eight contributions on various aspects of regenerative medicine in stroke recovery.

Within minutes of a stroke, millions of brain neurons perish (Saver, 2006; Overgaard, 2014). Although stroke-related cell death is irreversible, stem cell therapy may be of assistance (Wang et al., 2012; Rikhtegar et al., 2019). In light of this, Singh et al. conducted a critical review of the post-stem cell transplantation pattern of stroke recovery. The authors summarized the current state of stem cell therapy in stroke in pre-clinical and clinical settings. They put a strong emphasis on making the therapy translational. They also looked at a possible scenario for how stem cells might work to reverse symptoms. They also reviewed the clinical parameters that must be addressed before stem cell therapy for stroke is established, such as the type and amount of stem cells to be given, when to give them, whether dose-boosters are needed, how to administer them, etc. Without a doubt, stem cell therapy for stroke recovery is still in its infancy and requires more study and clinical trials before it can be made available in clinics. In this context, exogenous neurogenesis enhancement seems like a good way to help stroke patients, and Balseanu et al. looked into this.

Balseanu et al. descriptions of the regulation of neurogenesis following electric stimulation are noteworthy, particularly with regard to the impact of age as well as the molecular mechanisms affecting sensorimotor skills. In particular, the authors provide extensive data on how electric stimulation may stimulate neurogenesis in older animals, enhancing the brain's self-repair ability and improving behavioral recovery after focal ischemia. In summary, the authors addressed how two sessions of electrical non-convulsive stimulation on days 7 and 24 following middle cerebral artery occlusion (MCAO) enhanced functional recovery of spatial long-term memory (T-maze), but not on the rotating pole or inclined plane. Interestingly, they discovered that electric stimulation exacerbated the asymmetric sensorimotor deficiency. Increased doublecortin-positive cell numbers in the infarcted hemisphere's dentate gyrus and sub-ventricular zone, as well as the presence of a significant number of neurons expressing tubulin beta III in the infarcted area after electric stimulation was observed. This proved the neurogenic potential of electric stimulation therapy.

Nanoparticle technology has enabled the development of neuroprotective drugs for stroke recovery (Ahmad et al., 2019; Prakash et al., 2021a,b). In order to achieve enhanced neuroprotection, Rathore et al. infused collagen nanoparticles encapsulated with Silymarin into rats prior to middle cerebral artery occlusion. Remarkably, they discovered that Silymarin encased in collagen nanoparticles given intravenously for 7 days prior to MCAO treatment reduced infarct size while improving functional outcome. On this note, Naqvi et al. reviewed various nanoformulations for neuroprotective drug delivery. The authors reviewed the role of nanocarrier systems, including liposomes, micelles, solid lipid nanoparticles (SLNPs), dendrimers, and nanoemulsions, for the delivery of various neurotherapeutic agents. In addition, the mechanism of action and nanoformulation of various neuroprotective agents, including curcumin, edaravone, and nerve growth factors, have been discussed. Nanoparticle-based drugs for stroke still face a number of challenges, particularly those relating to the design and synthesis of novel nanoparticles with site-specific effects (Alkaff et al., 2020; Dong et al., 2020). Their mode of delivery, timing of delivery, and how to track them in patients are additional challenges (Wu et al., 2020). However, as our understanding of the aforementioned questions deepens so will the efficacy of treating patients with ischemic stroke.

Of particular note is that Chen et al. focused on a cancer patient who was prone to thrombotic events, often referred to as Trousseau syndrome (Ikushima et al., 2016). They discussed the case of a 55-year-old man who underwent surgery to remove his lower esophageal cancer but received no further treatment. He was brought into the hospital's emergency room 3 months later with multiple cerebral infarctions. The patient underwent IVT with tissue plasminogen activator (rtPA), and the symptoms subsided by the end of the procedure. However, after treatment, he did suffer from repeated cerebral infarctions and bleeding. The clinical course of this case indicates that it is important to carefully consider whether thrombolysis with rtPA is appropriate in the acute phase of cerebral infarction complicated by Trousseau syndrome. On the other hand, Yang et al. investigated the efficacy and safety of hybrid surgery, which is a surgical method for symptomatic chronic complete internal carotid artery occlusion (ICAO). According to the authors, hybrid surgery may be safe and effective for patients with symptomatic chronic complete ICAO. Also notable is the Hu et al. study that presents a rare instance of spontaneous intraventricular hemorrhage-induced fulminant Guillain-Barré Syndrome. Flaccid paralysis is a hallmark of the immune-mediated acute inflammatory peripheral polyneuropathy known as Guillain-Barré Syndrome (van den Berg et al., 2014; Willison et al., 2016). There have been a few cases where GBS has been linked to head trauma or neurosurgery, but intraventricular hemorrhage has never been mentioned. Hence, the Hu et al. study appears to be the first to identify fulminant Guillain-Barré Syndrome following spontaneous intraventricular hemorrhage.

Finally, yet importantly, corticofugal projection neurons are neurons that transmit excitatory input to the subcerebral nuclei and connect the cerebral cortex and sub-cortex (Lodato et al., 2015; Zhu et al., 2016). However, no corticofugal projection neurons-specific surface markers were identified for the purification. Along these lines, Sunohara et al. propose miRNA124-3p as a marker for identifying live corticofugal projection neurons-like cells derived from mouse ESC-derived cortical neurons.

In brief, we wish to emphasize aspects of the above studies which may help in the development of new stem cell and/or nanomedicine-based-therapies in addition to surgical interventions, as well as in understanding the pathophysiological mechanisms leading to stroke (and its associated commodities). We expect that this specialized issue will offer researchers useful information that will motivate them to carry out more investigations in this fascinating field.

## Author contributions

The editorial was written by SR. The editorial was edited by HA, VM, and AP. The article's submission was reviewed and approved by all authors.
